# Impact of tumour characteristics and cancer treatment on cerebrovascular mortality after glioma diagnosis: Evidence from a population-based cancer registry

**DOI:** 10.3389/fonc.2022.1025398

**Published:** 2022-12-09

**Authors:** Kai Jin, Paul M. Brennan, Michael T. C. Poon, Jonnie D. Figueroa, Cathie L. M. Sudlow

**Affiliations:** ^1^ Usher Institute, University of Edinburgh, Edinburgh, United Kingdom; ^2^ Brain Tumour Centre of Excellence, Cancer Research United Kingdom Edinburgh Centre, University of Edinburgh, Edinburgh, United Kingdom; ^3^ Translational Neurosurgery, Centre for Clinical Brain Sciences, University of Edinburgh, Edinburgh, United Kingdom

**Keywords:** brain tumours, cerebrovascular mortality, risk factors, tumour aggressiveness, cancer treatment, radiotherapy, epidemiologyAbstract (250)

## Abstract

**Objective:**

We aimed to examine brain tumour grade, a marker of biological aggressiveness, tumour size and cancer treatment are associated with cerebrovascular mortality among patients with malignant glioma, the most common and aggressive type of brain tumour.

**Methods:**

We conducted a retrospective, observational cohort study using the US National Cancer Institute’s state and regional population-based cancer registries. We identified adult patients with glioma in 2000 to 2018 (N=72,916). The primary outcome was death from cerebrovascular disease. Cox regression modelling was used to estimate the associations with cerebrovascular mortality of tumour grade, tumour size and treatment (surgery, radiotherapy, chemotherapy), calculating hazard ratios (HR) adjusted for these factors as well as for age, sex, race, marital status and calendar year.

**Results:**

Higher grade (Grade IV vs Grade II: HR=2.47, 95% CI=1.69-3.61, p<0.001) and larger brain tumours (size 3 to <6 cm: HR=1.40, 95% CI=1.03 -1.89, p<0.05; size ≥ 6 cm: HR=1.47, 95% CI=1.02-2.13, p<0.05 compared to size < 3cm) were associated with increased cerebrovascular mortality. Cancer treatment was associated with decreased risk (surgery: HR= 0.60, p<0.001; chemotherapy: HR=0.42, p<0.001; radiation: HR= 0.69, p<0.05). However, among patents surviving five years or more from cancer diagnosis radiotherapy was associated with higher risk of cerebrovascular mortality (HR 2.73, 95% CI 1.49-4.99, p<0.01).

**Conclusion:**

More aggressive tumour characteristics are associated with increased cerebrovascular mortality. Radiotherapy increased risk of cerebrovascular mortality five-year after cancer diagnosis. Further research is needed to better understand the long-term cardiovascular consequences of radiation therapy, and whether the consequent risk can be mitigated.

## Introduction

Cerebrovascular diseases, including stroke, are the commonest life threatening and disabling neurological disorders. Higher mortality rates from stroke have been reported for cancer patients compared with the general population (1), particularly for brain tumour patients who have over 7 times higher risk of fatal stroke than that of the general population, one of the highest relative risk among all cancer types (2). The mechanisms of stroke in cancer patients are complex. They include cancer-mediated hypercoagulability that increases the risk of thromboembolic events as well as cancer treatment-associated thrombosis (1, 3, 4). Previous studies have reported late-occurring stroke associated with radiotherapy in childhood cancer survivors and in head and neck cancer patients (5, 6). In patients with brain tumour specifically, the increased risk of stroke may result from tumour-related factors including systemic effects of the underlying tumour, direct tumour compression or infiltration, or cancer therapies, including cranial surgery related complications and radiation-induced vasculopathy (1, 3, 6–9). In a study of patients with childhood brain tumour, over half of subsequent strokes occurred 5 years or longer from their diagnosis (8).

Many brain tumours are associated with significant morbidity and mortality. The high cerebrovascular mortality rate in patients with brain tumours, including both benign and malignant tumours, should therefore prompt consideration of preventive intervention. This could improve survival outcomes, particularly in the subset of longer-term survivors (10), but also maximise the quality of life of many other patients. In some brain tumour patients, cerebrovascular disease that results in a significant neurological deficit curtails access to chemotherapy, which might otherwise be effective in extending patient survival.

Despite the high risk for fatal stroke outcome, it remains largely unknown which tumour and treatment factors are associated with cerebrovascular mortality in patients with brain tumours. Although cancer treatment associated cardiovascular toxic effects, particularly radiation -induced cerebrovascular mortality has been well established in the long-term survivors of childhood brain tumours (11), cerebrovascular mortality risk among adult-onset brain tumours has not been well characterised. There is a lack of long-term safety data regarding cerebrovascular mortality in adults with brain tumours, particularly in those with low-grade tumour with a more favourable oncologic outcome. Most previous studies were limited to childhood cancer survivors, clinical case series or examining single factors with small sample sizes (6, 12–14). A better understanding of tumour- and treatment-related factors associated with cerebrovascular mortality should provide important insights for identification of high-risk groups and improved medical management.

With a focus on glioma, the most common adult primary brain tumour, we aimed to examine to what extent brain tumour grade, a marker of biological aggressiveness, tumour size and treatment are each associated with cerebrovascular mortality using population-based data from the US. We also examined the effect of tumour treatment on cerebrovascular mortality during different time periods after diagnosis.

## Methods

### Study design and data source

We followed the Strengthening the Reporting of Observational Studies in Epidemiology (STROBE) guidelines (15). We used data from state and regional population-based cancer registries from the Surveillance, Epidemiology, and End Results (SEER) database (SEER 18 registry) (16). Approval to access the SEER data was granted by the US National Cancer Institute (NCI). In brief, SEER is a population-based incident tumour registries from geographically distinct regions in the USA, covering 28% of the US population, including incidence, survival, and treatment data. The SEER database is representative of the population of the US, and this has been validated by external studies (17). The SEER registry incudes socio-demographic information such as sex, age at diagnosis, race/ethnicity, marital status, and year of diagnosis, tumour characteristics including stage of disease, grade, size, cancer treatment (surgery, chemotherapy, radiotherapy), and survival status.

### Study population

We identified adults (≥18 years) diagnosed with glioma between 2000 and 2018 from SEER. Gliomas were classified based on histological and molecular type (18). We classified patients with glioma using International Classification of Diseases 10th revision (ICD-10) codes C700-C729. We used the International Classification of Diseases for Oncology third edition (ICD-O-3) codes to group gliomas following the definitions from the Central Brain Tumour Registry of the United States (CBTRUS) (19). Inclusion criteria required cases to have been actively followed up, not previously diagnosed with a primary cancer, and to have pathologic confirmation of the glioma diagnosis.

### Exposures

The primary exposures include tumour grade, tumour size and treatments. Based on World Health Organisation (WHO) criteria, glioma is classified into four grades, with higher grade indicating increasing tumour aggressiveness (20): Grade I incudes pilocytic astrocytoma, Grade II includes low grade diffuse astrocytoma, Grade III includes anaplastic astrocytoma and Grade IV includes the most aggressive and malignant glioblastoma (GBM). Histology codes follow the definitions from the Central Brain Tumour Registry of the United States (CBTRUS) (16). Although pilocytic astrocytoma (Grade I) is classified as a non-malignant tumour by the WHO, this histology has been historically classified as malignant for mandatory US cancer registry reporting (21). Tumour sizes were grouped as <3cm, 3 to <6 cm and ≥6cm. Treatment variables included surgery intervention (having surgery or not having surgery/unknown); radiation therapy (radiation given/no radiation given (no/unknown/refused/recommended, unknown if administered) and chemotherapy (yes and no/unknown).

### Outcome

The outcome of interest was primary cause of death from cerebrovascular disease using ICD-10 codes (I60-69), including the following subtypes: non-traumatic intracranial haemorrhage, cerebral infarction, occlusion and stenosis of cerebral of precerebral vessels without infarction, other cerebrovascular diseases, and sequelae of cerebrovascular disease (late effect) (16).

### Covariates

We included the following covariates in our analysis: age at diagnosis, sex (male, female), race/ethnicity (non-Hispanic white, Hispanic, non-Hispanic Black, Asian/Pacific Island/American Indian/other), marital status (married/having partner, single/separated/divorced, unknown) and calendar years, diagnostic confirmation by microscopy or not.

### Statistical analysis

We performed descriptive analyses of baseline characteristics of patients with glioma, overall and according to glioma grade, summarising categorical variables as numbers and percentages per category. The Pearson’s Chi-squared test was used for comparison across glioma grades. We compared continuous variables across glioma grade subgroups using analysis of variance for normally distributed variables (summarized as means and standard deviations [SD]) or the Kruskal–Wallis test for non-normally distributed variables (summarized as medians and interquartile ranges). Deaths from other causes were censored at the time of death. Survival time from the date of diagnosis until date of death or last contact (December 31, 2018) were computed. We used multiple imputation with chained equations to impute missing values for tumour size. The imputation model included all variables in the **Table 1**. Kaplan-Meier methods with log-rank tests were used to assess the differences in cerebrovascular mortality in gliomas patients, comparing cerebrovascular cause-specific mortality between groups by the log-rank test. Cause-specific multivariable Cox regression models to estimate hazard ratios (HRs) and 95% confidence intervals (CIs) for the association between cerebrovascular mortality and tumour grade (II-IV) were used, tumour size (<3cm, 3 to <6 cm, ≥6cm), and treatment status (surgery yes/no, radiation therapy yes/no, chemotherapy yes/no). We also calculated HRs for the association between cancer treatment and cerebrovascular mortality stratified by different survival periods (<1, 1 to 5 & ≥5 years) after cancer diagnosis: survived within 1 year, 1-5 years or survived after 5 years after the diagnosis.

**Table 1:** Characteristics of the study cohort SEER 2000-2018.

Characteristics	[ALL]	Grade 1	Grade 2	Grade 3	Grade 4
Number of patients (% of total)	N=72916	N=1754 (2.4)	N=10673 (14.6)	N=16303 (22.4)	N=44186 (60.6)
**Sex**					
** Female**	31511 (43.2%)	823 (46.9%)	4667 (43.7%)	7454 (45.7%)	18567 (42.0%)
** Male**	41405 (56.8%)	931 (53.1%)	6006 (56.3%)	8849 (54.3%)	25619 (58.0%)
**Age, median (IQR)**^a^, years	59.0 [46.0;70.0]	31.0 [22.0;44.0]	46.0 [34.0;59.0]	50.0 [37.0;64.0]	64.0 [55.0;73.0]
**Age group**					
** ≤65 years**	47091 (64.6%)	1659 (94.6%)	8936 (83.7%)	12623 (77.4%)	23873 (54.0%)
** >65 years**	25825 (35.4%)	95 (5.42%)	1737 (16.3%)	3680 (22.6%)	20313 (46.0%)
**Year of diagnosis**					
** 2000-2004**	17795 (24.4%)	454 (25.9%)	3131 (29.3%)	4072 (25.0%)	10138 (22.9%)
** 2005-2009**	18892 (25.9%)	433 (24.7%)	3015 (28.2%)	4287 (26.3%)	11157 (25.3%)
** 2010-2014**	20023 (27.5%)	509 (29.0%)	2603 (24.4%)	4523 (27.7%)	12388 (28.0%)
** 2015-2018**	16206 (22.2%)	358 (20.4%)	1924 (18.0%)	3421 (21.0%)	10503 (23.8%)
**Race/ethnicities**					
** Non-Hispanic White**	55510 (76.1%)	1207 (68.8%)	7777 (72.9%)	11721 (71.9%)	34805 (78.8%)
** Hispanic (All Races)**	8669 (11.9%)	266 (15.2%)	1519 (14.2%)	2256 (13.8%)	4628 (10.5%)
** Non-Hispanic Black**	4385 (6.01%)	149 (8.49%)	642 (6.02%)	1096 (6.72%)	2498 (5.65%)
** Asian/Pacific Island/American Indian/other**	4352 (5.97%)	132 (7.53%)	735 (6.89%)	1230 (7.54%)	2255 (5.10%)
**Marital status**					
** Married/Partner**	43306 (59.4%)	645 (36.8%)	5957 (55.8%)	9061 (55.6%)	27643 (62.6%)
** Single/Separated/Divorced**	26618 (36.5%)	1021 (58.2%)	4261 (39.9%)	6452 (39.6%)	14884 (33.7%)
** Unknown**	2992 (4.10%)	88 (5.02%)	455 (4.26%)	790 (4.85%)	1659 (3.75%)
**Tumour size:**					
** < 3 cm**	16206 (22.2%)	684 (39.0%)	2838 (26.6%)	4680 (28.7%)	8004 (18.1%)
** 3 to < 6 cm**	41026 (56.3%)	833 (47.5%)	5523 (51.7%)	8304 (50.9%)	26366 (59.7%)
** ≥ 6 cm**	15684 (21.5%)	237 (13.5%)	2312 (21.7%)	3319 (20.4%)	9816 (22.2%)
**Diagnostic confirmation**					
** Microscopically Confirmed**	66301 (90.9%)	1711 (97.5%)	10129 (94.9%)	13539 (83.0%)	40922 (92.6%)
** Not Microscopically Confirmed**	6168 (8.46%)	40 (2.28%)	498 (4.67%)	2672 (16.4%)	2958 (6.69%)
** Unknown**	447 (0.61%)	3 (0.17%)	46 (0.43%)	92 (0.56%)	306 (0.69%)
**Surgery performed:**					
** Yes**	52763 (72.4%)	1547 (88.2%)	7540 (70.6%)	10472 (64.2%)	33204 (75.1%)
** No**	20153 (27.6%)	207 (11.8%)	3133 (29.4%)	5831 (35.8%)	10982 (24.9%)
**Radiation**					
** Yes**	45396 (62.3%)	271 (15.5%)	4938 (46.3%)	8962 (55.0%)	31225 (70.7%)
** None/Unknown/Refused**	27520 (37.7%)	1483 (84.5%)	5735 (53.7%)	7341 (45.0%)	12961 (29.3%)
**Chemotherapy**					
** Yes**	36394 (49.9%)	99 (5.64%)	3608 (33.8%)	6627 (40.6%)	26060 (59.0%)
** No/Unknown**	36522 (50.1%)	1655 (94.4%)	7065 (66.2%)	9676 (59.4%)	18126 (41.0%)
**Survival months**	12.0 [4.00;35.0]	86.0 [34.0;149]	44.0 [12.0;105]	30.0 [8.00;87.0]	7.00 [3.00;16.0]
**Survival Months, median (IQR)** ^a^					
** < 1 year**	35858 (49.2%)	181 (10.3%)	2607 (24.4%)	5024 (30.8%)	28046 (63.5%)
** 1 to < 2 year**	13544 (18.6%)	145 (8.27%)	1309 (12.3%)	2315 (14.2%)	9775 (22.1%)
** 2 to < 5 years**	10777 (14.8%)	338 (19.3%)	2191 (20.5%)	3318 (20.4%)	4930 (11.2%)
** ≥5 years**	12737 (17.5%)	1090 (62.1%)	4566 (42.8%)	5646 (34.6%)	1435 (3.25%)
**Vital status**					
** Alive**	53975 (74.7%)	5293 (49.6%)	7470 (45.8%)	4419 (10.0%)	18661 (25.6%)
** Dead**	18277 (25.3%)	5380 (50.4%)	8833 (54.2%)	39767 (90.0%)	54255 (74.4%)
**Death from cerebrovascular disease(n)**	377	6	68	124	179

Glioma was classified into four grades based on WHO criteria higher grade indicates increasing tumour aggressiveness. Grade I incudes pilocytic astrocytoma, Grade II includes low grade diffuse astrocytoma, Grade III includes anaplastic astrocytoma and Grade IV includes the most aggressive and malignant glioblastoma multiforme. Grade I classified as a non-malignant tumour by WHO, this histology has been historically classified as malignant for mandatory US cancer registry reporting.

a Number presented in median/Interquartile range (IQR).

We restricted survival analyses to those with grade II-IV gliomas because of small numbers of cerebrovascular deaths among patients with grade I glioma (N=6) and lack of events in some subgroups. Univariable analyses were performed and variables with p-value<0.10 were retained in the final multivariable regression model, which included age, sex, ethnicity/race, marital status, calendar year, tumour grade, tumour size, and treatment status. We assessed the potential for effect modification by age group (18-65 years, >65 years), sex, and race/ethnicity by including interaction terms between the exposures (tumour grades, tumour size and treatment) and these variables. Where we found a significant interaction, we conducted subgroup analyses to demonstrate the different HRs for relevant subgroups according to age, sex and/or ethnicity.

In sensitivity analyses to assess the robustness of our results, we repeated the above analyses with the study period limited to after 2005 to assess whether the introduction of adjuvant chemotherapy treatment from 2005 influenced the results (22, 23). To reduce the chance of reverse causality, we also performed landmark analyses, with follow-up commencing 1 month after cancer diagnosis, thereby excluding patients with an event of death from cancer or cerebrovascular disease within one month of diagnosis (23). Associations and interactions were considered statistically significant when the two-sided p value was < 0.05. We prepared and analysed data using R version 4.0.

### Data availability

Anonymized data not published within this article will be made available by request from any qualified investigator. No additional informed consent was required as there was no individual patient involvement.

## Results

### Cohort characteristics

We identified 91,655 patients diagnosed with a malignant brain tumour in SEER between 2000 and 2018. There were 72, 916 cases of glioma with a total follow-up time of 266,491 person-years (median survival=12 months [IQR 4, 35]; 56.8% males) (**Table 1**). The derivation of the final cohort is illustrated in **Supplemental Figure 1**. Most patients were aged ≤ 65 years at diagnosis, especially for lower grade tumours (Grade 1: 94.6%; Grade II 83.7%; Grade III 77.4%; Grade IV: 54.0%). The majority of patients had grade IV glioma, including the most aggressive glioblastoma (60.6%). A total of 377 patients died from cerebrovascular disease during the study period (**Table 2**). Over half of the cerebrovascular deaths occurred in those diagnosed ≤65 years and 80% occurred among those with higher grades: Grade III (n=124, 32.9%) and Grade IV gliomas (n=179, 47.5%).

**Table 2:** Cerebrovascular death in patients with gliomas SEER 2000-2018.

Characteristics	Cerebrovascular death N=377
**Sex**	
** Female**	169 (44.8%)
** Male**	208 (55.2%)
**Age, median (IQR) a, years**	64.0 [53.0;76.0]
**Age group**	
** ≤65 years**	199 (52.8%)
** >65 years**	178 (47.2%)
**Year of diagnosis**	
** 2000-2004**	112 (29.7%)
** 2005-2009**	119 (31.6%)
** 2010-2014**	95 (25.2%)
** 2015-2018**	51 (13.5%)
**Race/ethnicities**	
** Non-Hispanic White**	266 (70.6%)
** Hispanic (All Races)**	51 (13.5%)
** Non-Hispanic Black**	41 (10.9%)
** Other ethnic groups**	19 (5.04%)
**Marital status**	
** Married/Partner**	199 (52.8%)
** Single/Separated/Divorced**	154 (40.8%)
** Unknown**	24 (6.37%)
**Glioma grade**	
** Grade 1**	6 (1.59%)
** Grade 2**	68 (18.0%)
** Grade 3**	124 (32.9%)
** Grade 4**	179 (47.5%)
**Tumour size**	
** < 3 cm**	79 (21.0%)
** 3 to < 6 cm**	229 (60.7%)
** ≥ 6 cm**	69 (18.3%)
**Survival months (IQR)**	8.00 [0.00;40.0]
**Survival time**	
** < 1 year**	204 (54.1%)
** 1 to < 2 years**	50 (13.3%)
** 2 to < 5 years**	53 (14.1%)
** ≥5 years**	70 (18.6%)
**Surgery**	
** Surgery**	210 (55.7%)
** No surgery**	167 (44.3%)
**Radiation**	
** None/Unknown/Refused**	232 (61.5%)
** Radiation given**	145 (38.5%)
**Chemotherapy**	
** Yes**	92 (24.4%)
** No/Unknown**	285 (75.6%)

### Factors associated with cerebrovascular mortality in patients with glioma

**Figure 1:**
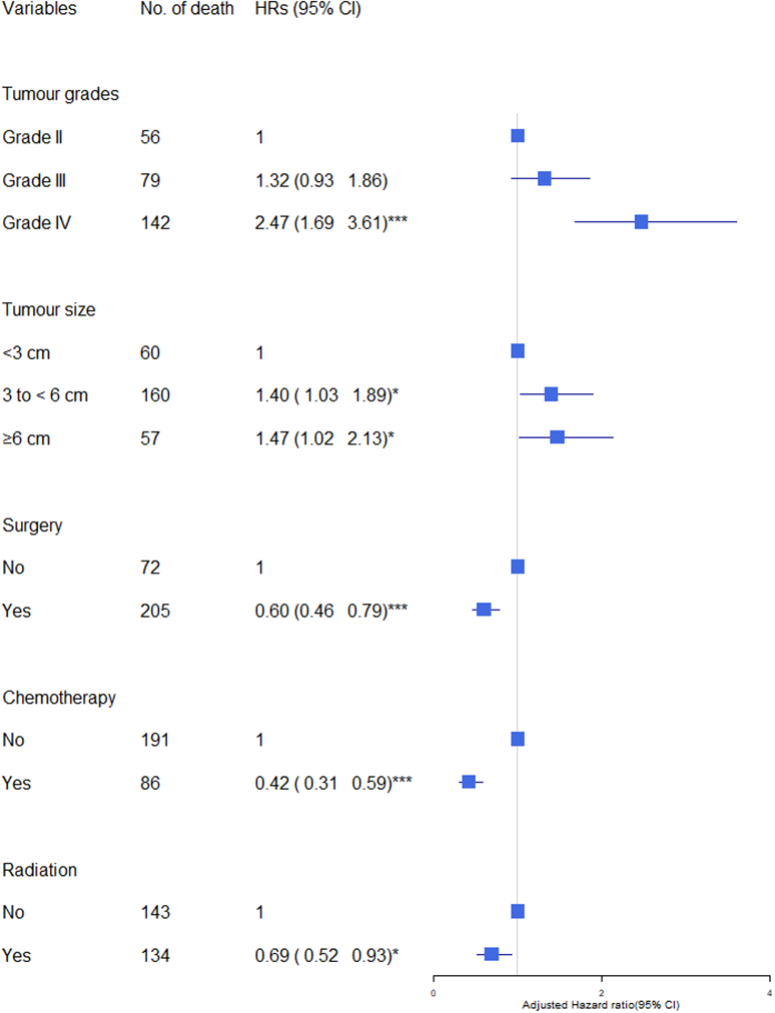
Association of tumour characteristics and cancer treatment with cerebrovascular mortality in glioma patients. The hazard ratios (HRs) were calculated using cox regression model and adjusted for variable shown, age, sex, race/ethnicity, marital status and calendar years. CI, Confidence intervals. Significance code: *** p<0.001, * p<0.05.

We observed increased cerebrovascular mortality in glioma patients with higher grade (Grade IV: HR=2.47, 95% CI=1.69-3.61 compared to Grade II, p<0.001), and those with larger brain tumours (size 3 to <6 cm: HR=1.40, p<0.05; 6 to <9 cm: HR=1.47, p<0.05 compared to size < 3cm) after adjusted by age, sex, race/ethnicity, marital status and calendar years(**Figure 1**). Having cancer treatment was associated with decreased risk: surgery (yes VS no: HR= 0.60; p<0.001), radiation (yes VS no: HR= 0.69, p<0.001), chemotherapy (yes VS no: HR=0.42, p<0.001).

**Figure 2:**
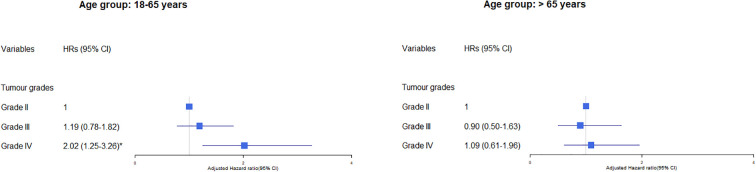
Association of tumour grade with cerebrovascular mortality in glioma patients by age group. The hazard ratios (HRs) were calculated using cox regression model and adjusted for variable shown, age, sex, race/ethnicity, marital status and calendar years. The reference group was those who did not receive the treatment: surgery, chemotherapy or radiation. CI, Confidence intervals. Significance code: * p<0.01.

We found a significant interaction (p<0.001) between tumour grade and age group with no evidence of interaction for sex and ethnic group. In subgroup analyses of the effects of tumour grade on cerebrovascular mortality by age group, the relative risk of cerebrovascular mortality was significantly higher in younger than older patients with grade IV (aHR grade IV versus grade II in patients aged 18-65 years: 2.02, 95% CI 1.24-3.26, and in patients aged > 65 years: 1.09, p5% CI 0.61-1.96) (**Figure 2**). We found no evidence of significant interaction between tumour size, cancer treatment and sex, ethnic group, or age.

### Effects of cancer treatment on cerebrovascular mortality by different follow-up periods

**Figure 3:**
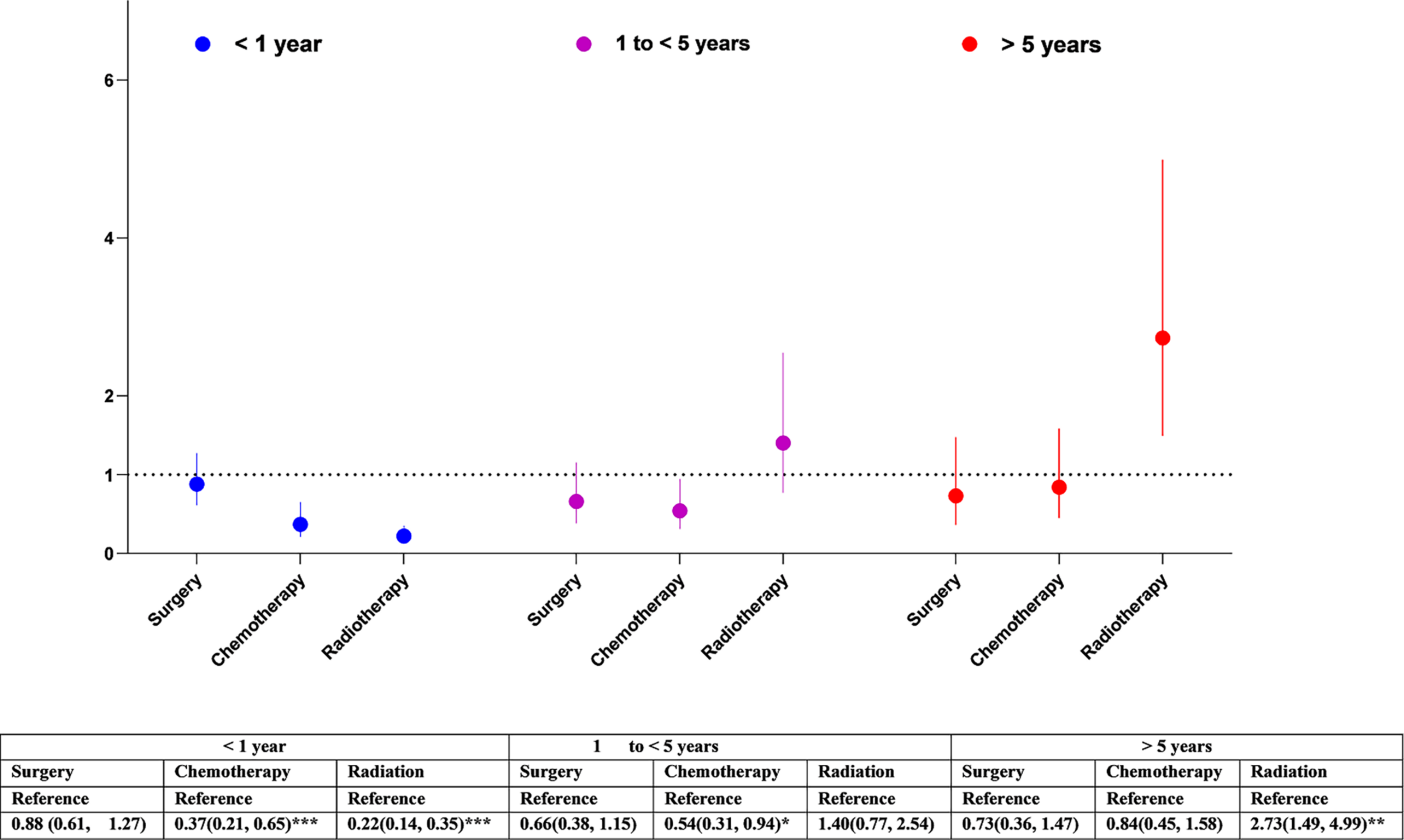
Association of cancer treatment with cerebrovascular mortality in glioma patients by different follow-up periods. The hazard ratios (HRs) were calculated using cox regression model and adjusted for sex, race/ethnicity, marital status and calendar years, tumour size, cancer treatments. The reference groups were those who did not receive the treatment: surgery, chemotherapy, or radiation. CI, Confidence intervals. Significance code: *** p<0.001, ** p<0.01, * p<0.05.

Overall, having cancer treatment was associated with decreased risk of cerebrovascular mortality: (surgery: HR= 0.65, 95% CI 0.46-0.79, p<0.001; chemotherapy: HR=0.42, 95% CI 0.31-0.59, p<0.001; radiation: HR= 0.69, 95% CI 0.52-0.93, p<0.05) (**Figure 1**). The effects of each type of treatment on cerebrovascular mortality in different survival periods are shown in **Figure 3**. The associations of surgery and chemotherapy with cerebrovascular mortality were qualitatively similar for analyses restricted to the first 5 years after diagnosis and for patients surviving more than 5 years from their cancer diagnosis. By contrast, while radiotherapy was associated with a reduced risk of cerebrovascular mortality in the first year (HR=0.22, 95% CI 0.14-0.35, p<.0001), in glioma patients who survived more than 5 years from their cancer diagnosis, patients having radiotherapy had an almost 3-fold risk of cerebrovascular mortality (HR 2.73, 95% CI 1.49-4.99, p<0.01) (**Figure 3**). We repeated the analysis in high grade (Grade 3 & 4) and low-grade group (Grade 2). Radiotherapy was associated with increased cerebrovascular mortality 5 years after diagnosis in both low grade (HR: 3.89, 95% CI 1.50-10.10, p<0.01) and high grade glioma patients (HR 2.47, 95% CI 1.09-5.58, p<0.5) (**Table 3**).

**Table 3:** Association of cancer treatment with cerebrovascular mortality in glioma patients by different follow-up periods stratified by low grade and high grade.

Low grade (Grade 2)
	Overall	< 1 year	1-5 years	>5 years
	HR (95% CI)	HR (95% CI)	HR (95% CI)	HR (95% CI)
**Surgery**
** No**	1	1	1	1
** Yes**	0.77 (0.43 1.38)	0.86 (0.25 2.89)	0.82 (0.34 1.97)	1.15 (0.41 3.26)
**Chemotherapy**
** No**	1		1	1
** Yes**	0.58 (0.30 1.15)	Insufficient number	1.00 (0.37 2.66)	0.61 (0.21 1.76)
**Radiation**
** No**	1	1	1	1
** Yes**	1.48 (0.84 2.60)	0.33 (0.07 1.56)	0.78 (0.31 1.98)	3.89 (1.50 10.10)**
**High grade tumour (Grade 3 &4)**
**Surgery**	
** No**	1	1	1	1
** Yes**	0.67 (0.51 0.89) **	0.90 (0.61 1.33)	0.63 (0.31 1.27)	0.77 (0.29 2.00)
**Chemotherapy**
** No**	1	1	1	1
** Yes**	0.45 (0.32 0.62) ***	0.41 (0.23 0.73) **	0.42 (0.22 0.82) *	1.07 (0.47 2.40)
**Radiation**
** No**	1	1	1	1
** Yes**	0.70 (0.52 0.93) *	0.21 (0.13 0.35) ***	1.80 (0.80 4.05)	2.47 (1.09 5.58) *

Association of cancer treatment with cerebrovascular mortality in glioma patients by different follow-up periods stratified by low and high grades. The hazard ratios (HRs) were calculated using cox regression model and adjusted for sex, race/ethnicity, marital status and calendar years, tumour size, cancer treatments. The reference groups were those who did not receive the treatment: surgery, chemotherapy, or radiation. CI, Confidence intervals. Significance code: *** p<0.001, ** p<0.01, * p<0.05.

### Sensitivity analysis

Similar results to those noted above were observed when we restricted analyses to those patients who survived one month from tumour diagnosis (**Supplemental Tables 1**, **2**). Broadly comparable results were also found among those diagnosed after 2005 (**Supplemental Tables 3**, **4**).

## Discussion

Our analysis of over 70,000 cases of primary gliomas using population-based data from SEER found that patients with higher grade, particularly the most aggressive gliomas, Grade IV, and larger gliomas were at increased risk of cerebrovascular mortality. Receiving cancer treatments was associated with lower risk for cerebrovascular mortality in patients surviving less than 5 years. However, radiation therapy significantly increased the risk of cerebrovascular mortality in longer-term survivors.

### Tumour-associated factors for cerebrovascular mortality in glioma patients

The association of high-grade glioma with cerebrovascular mortality suggests an important biological role for tumour aggressiveness in the risk of stroke. This is consistent with previous studies showing that patients with more advanced stage cancer, including lung, pancreatic, colorectal and gastric cancer, have increased risk of stroke (24). These findings suggest the biological plausibility of the relationship between stroke risk and aggressiveness of cancer types, implicating a systemic response to malignancy in stroke risk, for example from cancer-mediated hypercoagulability (24, 25). Glioma cells have inherent prothrombotic properties that secrete procoagulant proteins such as tissue factors, the principal initiator of coagulation that activates the clotting cascade. Tumour-induced hypercoagulability causes thrombus formation within the cerebral vasculature resulting in ischaemic hypoxia that leads to cerebral infarction (7, 26, 27). Similar mechanisms lead to the higher risk of venous thromboembolism (VTE) also seen in brain tumours, with the greatest risk in glioblastoma (27). The association of larger tumour size with higher cerebrovascular mortality risk may relate to reduced vascular perfusion from mass effect of tumour growth or to direct tumour invasion into surrounding brain tissue and vasculature (28, 29).

The findings of a particularly strong association between high grade glioma and cerebrovascular mortality in younger patients may further support the independent role of tumour aggressiveness-related hypercoagulation, because younger patients are relatively healthy and less likely than older individuals to have conventional stroke risk factors (30). The weaker association between tumour grade and cerebrovascular mortality in older patients may be attributable to unmeasured comorbidities or competing causes of mortality with increasing age. Advancing age is a risk factor for cardiovascular risk accompanied by the development of comorbidities such as hypertension and high cholesterol, as well as related to cerebral small vessel disease leading to stroke and cognitive decline (30).

### Treatment-related cerebrovascular mortality in glioma patients

Our findings showed that tumour treatment, particularly chemotherapy and radiation therapy, were associated with lower risk of cerebrovascular mortality in glioma patients surviving less than 5 years, while having radiation increased cerebrovascular mortality risk in those surviving more than 5 years. While cancer treatment reduces cancer activity, it has been recognised that treatment increases risk of fatal and non-fatal cardiovascular outcomes including stroke in brain tumour survivors (4, 31). Our findings of radiation-associated long-term increased risk of cerebrovascular mortality in adult gliomas survivors are consistent with previous investigations in long-term survivors of childhood cancers (14). Radiotherapy is used to reduce or prevent tumour growth. However, it may damage normal tissues, leading to irreversible chemical and biological changes, and resulting in cell death. Radiotherapy can accelerate atherosclerotic changes in the arterial wall, predisposing patients to vascular dysfunction and ischemic events (3, 14, 30). Radiation-induced vasculopathy can develop months to years after radiation therapy (32). There is evidence that cranial radiotherapy is associated with risk of late neurovascular events and stroke in younger brain tumour survivors (14). Radiotherapy at younger age and higher radiation does are risk factors for developing radiation vasculopathy (3, 6, 8). In older adults, increased risk of cerebrovascular mortality could be due to the combination effects from radiation and age-related atherosclerosis risk factors such as hypertension, hypercholesterolemia that are more prevalent in older adults (8, 32). For example, hypertension can directly damage arteries that predispose patient more vulnerable for stoke during and after brain treatment (33). there are increasing concerns of safe radiation regimes and efficacy in elderly patients for their tolerability and side effects (34). However, there is lack of enough evidence for optimal strategies and clinical guidelines in adult patients with GBM which often occurs in those aged over 65 years. These elderly patients with co-comorbidities are often excluded from clinical trials. Future prospective studies are needed with the aim of understanding the short-and long-term cerebrovascular complications of radiation therapy to guide for the optimal intervention (34, 35).

Radiation treatment remains the cornerstone of therapy for patients with brain tumour such as glioma. An awareness of the long-term risk of cerebrovascular mortality is not intended to detract from this standard care therapy, but rather to encourage incorporation of screening, mitigation and prevention methods where supported by an evidence-base (35). This may include mitigating cardiovascular risk factors. For patients with lower grade tumours and longer survival, consideration may be given to investigation of what schedules of radiotherapy reduce cerebrovascular risk whilst optimising tumour control (35).

### Strengths and limitations

Using the large population-based data, our study showed the important role of tumour aggressiveness and radiation therapy in cerebrovascular mortality in patients with gliomas. Our study is the largest and most comprehensive analysis to date of the associations of tumour characteristics and cancer treatments factors with cerebrovascular mortality in glioma patients, and used population-based data, enhancing the generalizability of our findings. Our findings provide important evidence for planning future clinical trials to understand the role of prophylaxis against arterial thrombosis and to guide clinical management. Our findings of long-term fatal cerebrovascular outcome from radiotherapy should enable better identification of groups of high-risk patients requiring surveillance and prevention of cerebrovascular complications, for example, assessing and treating cardiovascular risk factors such as hypertension. Importantly, long-term glioma survivors who have a more favourable oncologic prognosis may benefit the most from follow-up clinical screening and monitoring to improve their survival outcomes, particularly in those who have had radiotherapy.

The absolute number of glioma patients who died from cerebrovascular deaths are low in this population. However, this number may be under reported and not reflect the true burden of this disease, because in patients with a brain tumour a cerebrovascular event may not be considered as a cause of clinical deterioration. Prompt diagnosis of cerebrovascular diseases, including stroke is needed to achieve maximal functional recovery, and quality of life (36). This is especially important for patients with GBM where life expectancy is short. Stroke diagnosis is clinically challenging in patients with a brain tumour because of the overlapping symptoms. It can be difficult to distinguish tumour tissue from ischemic stroke on MR image in the setting of a pre-existing brain tumour (37). One study showed the initial clinical diagnosis was correct in only 45% of ischemia stroke episodes in patients with primary brain tumour (8), reflecting the difficulty of diagnosing stroke and possibility of undetected cases. Further, in addition to the traditional risk factors, stroke in cancer patients may involve complex underlying biology that remains poorly understood (1). Advances in molecular and gene profiling of brain tumour may have a role to understand the complex phenomenon, and how to mitigate stroke risk. Our study, by far, is the largest study to examine this under-research but clinically important issue, may pave the way for further research.

Our study has limitations. First, it was retrospective in nature, and lacked granular details of stroke diagnosis, including stroke subtypes. Future prospective studies are needed with more granular detail of stroke diagnosis such as stroke subtypes, timing of the event, biomarkers by including neuroimaging and laboratory data to improve the diagnosis of stroke and determine the cause of stroke. Second, we did not have baseline cardiovascular risk factors (i.e., hypertension, diabetes) and/or cardiovascular disease (i.e, coronary heart disease, atherosclerosis). However, our analysis in the younger age group who were relatively healthy has shown the strong association between tumour aggressiveness and cerebrovascular mortality. In addition, a population-based cohort study using UK Clinical Practice Research Datalink (CPRD) showed adjustment for shared CVD risk factors had little effect on CVD risk including stroke in adult survivors of multiple site-specific cancer including central nervous system (CNS) tumours compared with the general population (38). Third, our study is also limited by the lack of detailed data on cancer treatments, such as types and doses of cancer therapy and subsequent treatment. A more comprehensive approach by linking health data from various sources such as primary care data, anti-cancer therapies, image data will provide a better measurement to examine the factors that driver cerebrovascular mortality in brain cancer patients. Another limitation is the risk of misclassification of cause of death by use of death certificate information, although previous studies have reported acceptable validity (>80%) of cause of death using SEER data (39).

## Conclusions

More aggressive tumour characteristics are associated with increased cerebrovascular mortality. While receiving cancer treatments was associated with lower risk for cerebrovascular mortality, having radiation increased long-term fatal outcome for cerebrovascular disease. The complex interplay of putative risk and benefit from the tumour and its treatment underscore the need for further research. As early detection and more effective anticancer therapies extend the survival of cancer patients, avoiding treatment-related long-lasting fatal cerebrovascular outcome becomes increasingly vital. Knowledge of the risks can help clinicians be more vigilant for signs and symptoms of potential neurological complications and guide the management of long-term glioma survivors.

## Data availability statement

Publicly available datasets were analyzed in this study. This data can be found here: https://seer.cancer.gov/data/. Approval to access the SEER data was granted by the US National Cancer Institute (NCI). Data not published within this article will be made available by request from any qualified investigator.

## Ethics statement

Ethical review and approval was not required for the study on human participants in accordance with the local legislation and institutional requirements. Written informed consent for participation was not required for this study in accordance with the national legislation and the institutional requirements.

## Author contributions

KJ conceived and designed the study, analysed data, developed figures, interpreted data, and developed the draft of the manuscript. PB designed the study, interpreted data, contributed to the writing, reviewing and editing of the manuscript. MP contributed to data interpretation and reviewing the manuscript. CS conceived and designed the study, interpreted and verified data, contributed to the writing, reviewing and editing of the manuscript. JF conceived and designed the study, interpreted and verified data, contributed to the writing, reviewing and editing of the manuscript. All authors critically revised the manuscript for important intellectual content. All authors approved the manuscript to submit for publication. All authors contributed to the article and approved the submitted version.
