# Supplementary material for: Impact of tumour characteristics and cancer treatment on cerebrovascular mortality after glioma diagnosis: Evidence from a population-based cancer registry
Source: Front Oncol. 2022 Dec 9;12:1025398. doi: 10.3389/fonc.2022.1025398 (PMC9780584; doi:10.3389/fonc.2022.1025398)
Supplement: Supplementary file 1 [file DataSheet_1.docx]

# Supplemental Materials

# Impact of tumour characteristics and cancer treatment on cerebrovascular mortality after brain cancer diagnosis

Kai Jin, PhD^1,2^, Paul M Brennan, PhD^2,3^, Michael TC Poon, MSc^1,2,3^, Cathie LM Sudlow, DPhil^1,2*^, Jonine D Figueroa, PhD^1,2*^

*Contributed equally

^1^Usher Institute, University of Edinburgh, Edinburgh, UK

^2^Brain Tumour Centre of Excellence, Cancer Research UK Edinburgh Centre, University of Edinburgh, Edinburgh, UK

^3^Translational Neurosurgery, Centre for Clinical Brain Sciences, University of Edinburgh, Edinburgh, UK

**Supplemental methods**

# Supplemental Figures and Tables

### Supplemental Figure 1 Flowchart of the selection of the cohorts used in analyses

### Supplemental Table 1. Cerebrovascular death in patients with gliomas SEER 2000-2018

### Supplemental Table 2. Landmark analysis in cox proportional hazard regression model HRs for association between tumour grade, tumour size, cancer treatment and stroke mortality in glioma patients between 2000-2018

### Supplemental Table 3. Landmark analysis in cox proportional hazard regression model HRs for association between tumour grade, tumour size, cancer treatment and stroke mortality in glioma patients by age group between 2000-2018

### Supplemental Table 4 Cause-specific Cox proportional hazard regression model HRs for association between tumour grades, tumour size, cancer treatment and stroke mortality in gliomas patients after 2005

### Supplemental Table 5 Cause-specific Cox proportional hazard regression model HRs for association between tumour grades, tumour size, cancer treatment and stroke mortality in gliomas patients by age group after 2005

**Supplemental methods**

### Data source and study population

The SEER registry incudes socio-demographic information such as sex, age at diagnosis, race/ethnicity, marital status, and year of diagnosis, tumour characteristics including stage of disease, grade, size, cancer treatment (surgery, chemotherapy, radiotherapy), and survival status.

The SEER database is representative of the population of the US, and this has been validated by external studies^1^. We identified adults (≥18 years) diagnosed with glioma between 2000 and 2018 from SEER. Inclusion criteria required cases to have been actively followed up, not previously diagnosed with a primary cancer, and to have pathologic confirmation of the glioma diagnosis.

Gliomas were classified based on histological and molecular type Grade I incudes [pilocytic astrocytoma](https://www.sciencedirect.com/topics/medicine-and-dentistry/pilocytic-astrocytoma) , Grade II includes low grade diffuse astrocytoma, Grade III includes anaplastic astrocytoma and Grade IV includes the most aggressive and malignant glioblastoma (GBM). Histology codes follow the definitions from the Central Brain Tumour Registry of the United States (CBTRUS)^2^. Although pilocytic astrocytoma (Grade I) is classified as a non-malignant tumour by the WHO, this histology has been historically classified as malignant for mandatory US cancer registry reporting^3^. Inclusion criteria required cases to have been actively followed up, not previously diagnosed with a primary cancer, and to have pathologic confirmation of the glioma diagnosis.

### Statistical analysis

We performed descriptive analyses of baseline characteristics of patients with glioma, overall and according to glioma grade, summarising categorical variables as numbers and percentages per category. The Pearson’s Chi-squared test was used for comparison across glioma grades. We compared continuous variables across glioma grade subgroups using analysis of variance for normally distributed variables (summarized as means and standard deviations [SD]) or the Kruskal–Wallis test for non-normally distributed variables (summarized as medians and interquartile ranges).

We restricted survival analyses to those with grade II-IV gliomas because of small numbers of cerebrovascular deaths among patients with grade I glioma (N=6) and lack of events in some subgroups. Univariable analyses were performed and variables with p-value<0.10 were retained in the final multivariable regression model, which included age, sex, ethnicity/race, marital status, calendar year, tumour grade, tumour size, and treatment status. We assessed the potential for effect modification by age group (18-65 years, >65 years), sex, and race/ethnicity by including interaction terms between the exposures (tumour grades, tumour size and treatment) and these variables. Where we found a significant interaction, we conducted subgroup analyses to demonstrate the different HRs for relevant subgroups according to age, sex and/or ethnicity. We also performed sensitive analyses to assess the robustness of our results.

In sensitivity analyses to assess the robustness of our results, we repeated the above analyses with the study period limited to after 2005 to assess whether the introduction of adjuvant chemotherapy treatment from 2005 influenced the results^4,5^. To reduce the chance of reverse causality, we also performed landmark analyses, with follow-up commencing 1 month after cancer diagnosis, thereby excluding patients with an event of death from cancer or cerebrovascular disease within one month of diagnosis^5^. Associations and interactions were considered statistically significant when the two-sided p value was < 0.05. We prepared and analysed data using R version 4.0.

**Data availability**

Anonymized data not published within this article will be made available by request from any qualified investigator. No additional informed consent was required as there was no individual patient involvement.

### Supplemental Figure 1

Figure 1. Flowchart of the selection of the cohorts used in analyses
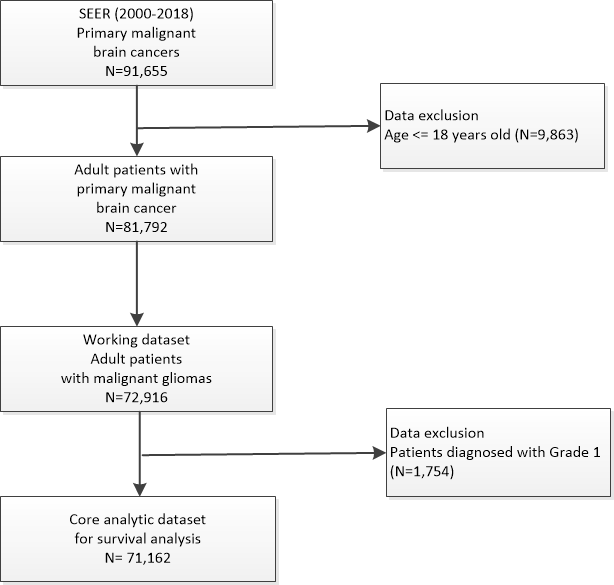


Flowchart of the selection of the cohorts used in the analyses. SEER: Surveillance, Epidemiology, and End Results(SEER) Program 18 Registries, the largest geographic coverage available approximately 27.8% of the US population. SEER data was linked to US. Mortality data.

### Supplemental Table 1 Cerebrovascular death in patients with gliomas SEER 2000-2018

| Characteristics | Cerebrovascular death  N=377 |
| --- | --- |
| Sex |  |
| Female | 169 (44.8%) |
| Male | 208 (55.2%) |
| Age, median (IQR) a, years | 64.0 [53.0;76.0] |
| Age group |  |
| ≤65 years | 199 (52.8%) |
| >65 years | 178 (47.2%) |
| Year of diagnosis |  |
| 2000-2004 | 112 (29.7%) |
| 2005-2009 | 119 (31.6%) |
| 2010-2014 | 95 (25.2%) |
| 2015-2018 | 51 (13.5%) |
| Race/ethnicities |  |
| Non-Hispanic White | 266 (70.6%) |
| Hispanic (All Races) | 51 (13.5%) |
| Non-Hispanic Black | 41 (10.9%) |
| Other ethnic groups | 19 (5.04%) |
| Marital status |  |
| Married/Partner | 199 (52.8%) |
| Single/Separated/Divorced | 154 (40.8%) |
| Unknown | 24 (6.37%) |
| Glioma grade |  |
| Grade 1 | 6 (1.59%) |
| Grade 2 | 68 (18.0%) |
| Grade 3 | 124 (32.9%) |
| Grade 4 | 179 (47.5%) |
| Tumour size |  |
| < 3 cm | 79 (21.0%) |
| 3 to < 6 cm | 229 (60.7%) |
| ≥ 6 cm | 69 (18.3%) |
| Survival months (IQR) | 8.00 [0.00;40.0] |
| Survival time |  |
| < 1 year | 204 (54.1%) |
| 1 to < 2 years | 50 (13.3%) |
| 2 to < 5 years | 53 (14.1%) |
| ≥5 years | 70 (18.6%) |
| Surgery |  |
| Surgery | 210 (55.7%) |
| No surgery | 167 (44.3%) |
| Radiation |  |
| None/Unknown/Refused | 232 (61.5%) |
| Radiation given | 145 (38.5%) |
| Chemotherapy |  |
| Yes | 92 (24.4%) |
| No/Unknown | 285 (75.6%) |

### Supplemental Table 2 Landmark analysis in Cox proportional hazard regression model HRs for association between tumour grades, tumour size, cancer treatment and cerebrovascular mortality in gliomas patients

| Characteristics | All cases |  |  |
| --- | --- | --- | --- |
|  | **Adjusted HRs** |  |  |
| Grades |  |  |  |
| Grade 2 | Reference |  |  |
| Grade3 | 1.32 (0.93 1.86) |  |  |
| Grade 4 | 2.47 (1.69 3.61)*** |  |  |
| Tumour size |  |  |  |
| <3 cm | Reference |  |  |
| 3 to < 6 cm | 1.40( 1.03 1.89)* |  |  |
| ≥6 cm | 1.47( 1.02 2.1)3* |  |  |
| Receiving surgery |  |  |  |
| No | Reference |  |  |
| Yes | 0.60( 0.46 0.79*** |  |  |
| Receiving radiation |  |  |  |
| No | Reference |  |  |
| Yes | 0.69 (0.52 0.93* |  |  |
| Receiving chemotherapy |  |  |  |
| No | Reference |  |  |
| Yes | 0.42 ( 0.31 0.59*** |  |  |
| Landmark analyses to those with follow-up commencing 1 month after cancer diagnoses to reduce the chance of reverse causality thereby excluding patients with an event (death or cerebrovascular disease event) within 1 months of cancer diagnosis. The hazard ratios were calculated using a cause-specific Cox proportional hazards regression model and adjusted by age, sex, ethnicity/race, marital status, calendar year. Significance codes: ‘***’ 0.001, ‘**’ 0.01, ‘*’ 0.05' | | |  |

### Supplemental Table 3 Landmark analysis in Cox proportional hazard regression model HRs for association between tumour grades and cerebrovascular mortality in gliomas patients by age group

| **Characteristics** | **Age ≤65 years** | **Age>65 years** |
| --- | --- | --- |
|  | **Adjusted HRs** | **Adjusted HRs** |
| **Grades** |  |  |
| **Grade 2** | Reference | Reference |
| **Grade3** | 1.19( 0.78 1.82) | 0.90 (0.50 1.63) |
| **Grade 4** | 2.02 ( 1.25 3.26)** | 1.09 (0.61 1.96) |
| Landmark analyses to those with follow-up commencing 1 month after cancer diagnoses to reduce the chance of reverse causality thereby excluding patients with an event (death or cerebrovascular disease event) within 1 months of cancer diagnosis. The hazard ratios were calculated using a cause-specific Cox proportional hazards regression model and adjusted by age, sex, ethnicity/race, marital status, calendar year, cancer treatment. Significance codes: ‘***’ 0.001, ‘**’ 0.01, ‘*’ 0.05' | | |

### Supplemental Table 4 Cause-specific Cox proportional hazard regression model HRs for association between tumour grade, tumour size, cancer treatment and cerebrovascular mortality in glioma patients diagnosed after 2005

| Characteristics | Overall | |
| --- | --- | --- |
|  | **Adjusted HRs(95% CI)** | |
| Grades |  | |
| Grade 2 | **Reference** | |
| Grade3 | 1.01 (0.59 1.72) | |
| Grade 4 | 2.25 (1.25 4.06)** | |
| Tumour size |  | |
| <=3 cm | **Reference** | |
| 3-6 cm | 1.82 ( 1.03 3.19)* | |
| 6-9cm | 1.07 (0.47 2.44) | |
| >9 cm | 2.11 (1.17 3.80)* | |
| Receiving radiation |  | |
| No | **Reference** | |
| Yes | 0.82 (0.51 1.31) | |
| Receiving chemotherapy |  | |
| No | **Reference** | |
| Yes | 0.33 (0.20 0.54)*** | |
| The hazard ratios were calculated using a cause-specific Cox proportional hazards regression model and adjusted by age, sex, ethnicity/race, marital status, calendar year. Signif. codes: ‘***’ 0.001, ‘**’ 0.01, ‘*’ 0.05 | |  |

### Supplemental Table 5 Cause-specific Cox proportional hazard regression model HRs for association between tumour grade and cerebrovascular mortality in glioma patients diagnosed after 2005 by age group

| **Characteristics** | **Age ≤65 years** | **Age>65 years** |
| --- | --- | --- |
|  | **Adjusted HRs(95% CI)** | **Adjusted HRs(95% CI)** |
| **Grades** |  |  |
| **Grade 2** | Reference | Reference |
| **Grade3** | 1.19( 0.78 1.82) | 0.90 (0.50 1.63) |
| **Grade 4** | 2.02 ( 1.25 3.26)** | 1.09 (0.61 1.96) |
| The hazard ratios were calculated using a cause-specific Cox proportional hazards regression model and adjusted by age, sex, ethnicity/race, marital status, calendar year, cancer treatment. Signif. codes: ‘***’ 0.001, ‘**’ 0.01, ‘*’ 0.05 | | |

### References

1. Lloyd S, Park HS, Decker RH, Wilson LD, Yu JB. Using the Surveillance, Epidemiology, and End Results database to investigate rare cancers, second malignancies, and trends in epidemiology, treatment, and outcomes. *Curr Probl Cancer.* 2012;36(4):191-199.

2. Jin K, Brennan PM, Poon MTC, Sudlow CLM, Figueroa JD. Raised cardiovascular disease mortality after central nervous system tumor diagnosis: analysis of 171,926 patients from UK and USA. *Neuro-Oncology Advances.* 2021;3(1).

3. Forjaz G, Barnholtz-Sloan JS, Kruchko C, et al. An updated histology recode for the analysis of primary malignant and nonmalignant brain and other central nervous system tumors in the Surveillance, Epidemiology, and End Results Program. *Neuro-Oncology Advances.* 2020;3(1).

4. Stupp R, Mason WP, van den Bent MJ, et al. Radiotherapy plus concomitant and adjuvant temozolomide for glioblastoma. *N Engl J Med.* 2005;352(10):987-996.

5. Anderson JR, Cain KC, Gelber RD. Analysis of survival by tumor response. *J Clin Oncol.* 1983;1(11):710-719.
